# Drivers of Veterans’ Healthcare Choices and Experiences with Veterans Affairs and Civilian Healthcare

**DOI:** 10.3390/healthcare12181852

**Published:** 2024-09-14

**Authors:** Sara Kintzle, Eva Alday, Aubrey Sutherland, Carl A. Castro

**Affiliations:** USC Suzanne Dworak-Peck School of Social Work, University of Southern California, 669 West 34th Street, Los Angeles, CA 90089, USA; ealday@usc.edu (E.A.); ajsuther@usc.edu (A.S.); cacastro@usc.edu (C.A.C.)

**Keywords:** veteran healthcare, civilian healthcare, veteran affairs, veterans, healthcare satisfaction

## Abstract

Background: Access to quality healthcare is essential to the well-being of U.S. veterans. Little is known about what drives veterans’ healthcare decisions. The purpose of this study was to explore factors that drive healthcare choices in veterans, and their experiences in the Veterans Health Administration (VA) and non-VA healthcare settings. Methods: Fifty-nine veterans participated in eight focus groups. Participants were asked to discuss factors that led to their choice of provider and their healthcare experiences. Thematic analysis was conducted to reveal themes around healthcare choices and use. Results: VA and non-VA users described positive experiences with care. VA users reported cost, quality, and ease of care as reasons for use. Non-VA healthcare setting users reported eligibility issues, negative perceptions of the VA, administrative bureaucracy, and lack of continuity of care as reasons they chose not to use VA care. VA users reported difficulty with red tape, continuity of care, limitations to gender specific care, and having to advocate for themselves. Conclusions: Veterans were satisfied with care regardless of where they received it. Experiences with civilian providers indicate that more could be done to provide veterans with choices in the care they receive. Despite positive experiences with the VA, the veterans highlighted needed improvements in key areas.

## 1. Introduction

Due to the nature of the physical and emotional stress often associated with military service, veterans experience health challenges at disproportionate rates when compared to their civilian counterparts [1,2,3]. Difficulties during transition can exacerbate this physical and emotional stress, contributing to the development of chronic health conditions [4]. Veterans experience chronic pain and musculoskeletal issues at higher rates and in more severity than non-veterans [5,6,7]. Research has also found veterans to have significant rates of traumatic brain injury, sleep issues, obesity, diabetes, stroke, post-traumatic stress disorder (PTSD), suicidality, depression, and alcohol and substance use disorders [2,8,9]. Physical and mental health morbidities are reported at even higher rates for veterans with combat experience. Combat veterans have showNon elevated rates of PTSD, chronic pain, stroke, alcohol and substance use disorder, and suicidality [9,10,11,12,13,14]. Access to quality healthcare is imperative to address the physical and mental health challenges experienced by veterans.

There have been significant strides over the past several decades in improving healthcare access, choices around utilization, and care experiences for U.S. veterans [15,16,17]. The Veterans Health Administration (VA) has gone through systematic quality improvement [18], while the 2018 Mission Act established a new veterans community care program that provides veterans with more healthcare options, with eligibility expanded to receive care through community providers [19]. Furthermore, passing of the Promise to Address Comprehensive Toxics (PACT) Act of 2022 expanded eligibility and access to VA healthcare and benefits for veterans exposed to burn pits and other toxic substances [20]. [Please note the authors recognize VHA is the appropriate abbreviation for the Veterans Health Administration. However, as veteran participants used the abbreviation VA, the authors have done so here to provide consistency throughout the article].

Veterans who use the VA overwhelming report satisfaction with their quality of care [21,22]. Despite this satisfaction, challenges around administrative bureaucracy, distance to VA care, wait times, and gender sensitive care continue to be reported by VA users [22,23]. A recent study on 3188 veterans living in Southern California found that 58% identified the VA as their primary source of healthcare [24], meaning that over two in five veterans received healthcare outside of the VA. The most prominent reasons for non-use of the VA by veterans in the study included perceptions that VA healthcare was not as good as healthcare elsewhere, too much trouble or red tape, and difficulty with access. 

Little focus has been given to what drives veterans’ healthcare decisions, particularly what factors impact their choices to use VA healthcare or seek care from civilian providers. Furthermore, while research has focused on the experience of veteran VA users, information on the experiences of veterans who use civilian care is scarce. The purpose of this study was to qualitatively explore factors that drive healthcare choice in veterans as well as their experiences in VA and non-VA healthcare settings. 

## 2. Materials and Methods

A descriptive-interpretive qualitative approach was used to explore the veterans’ choices, experiences, and decision-making process around their healthcare. Focus groups, guided by a semi-structured interview protocol, were used to collect data. This approach was utilized as it provides researchers with an in-depth understanding of specific experiences as well as reasons for observed patterns [25]. 

### 2.1. Focus Group Protocol

A focus group interview protocol was developed to guide the discussions to cover healthcare experiences and the factors that influenced the veterans’ decision-making around their choice of healthcare provider. This type of protocol was chosen as it provides guidance within the discussions while allowing natural and organic responses from the participants [26]. Questions included topics such as choice of healthcare provider, healthcare experiences, and satisfaction. Examples of questions include: (a) What are the factors that led to your decision to choose your primary source of healthcare? (b) What have your experiences been like using your primary source of healthcare? (c) What are the biggest challenges to accessing care? The interview protocol can be found in Table 1.

### 2.2. Recruitment 

All participants were veterans who had previously participated in a large needs assessment of Southern California veterans, answered questions about their healthcare use, and agreed to be contacted for future research [24]. Potential participants were sent an email invitation to participate in the study. The invitation contained a detailed study information sheet and a link to indicate their interest and availability to participate in a 45-minute focus group. Scheduling was designed based on participant availability and choice of healthcare provider, VA versus non-VA as their primary care provider. A total of eight online focus groups were scheduled, four VA and four non-VA groups. A breakdown of each can be found in Table 2. 

### 2.3. Participants

Focus group participants included 59 veterans in Southern California. The size of the focus groups varied but ranged from 4 to 10 participants (M = 7 participants per focus group). Most of the participants fell within 30–39 or 60–69 years of age (22% for each age group) with a mean age of 53 years for the entire sample (*SD* = 13.76). A majority of the sample identified as White (72%), followed by African American (15%) and Other (10%). Twenty-six percent reported being of Hispanic, Latino, or Spanish origin. Over half (59%) of the sample identified as male while 41 percent identified as female. Table 3 presents the sample characteristics by demographics. 

### 2.4. Procedures

Eligible participants were sent an email invitation that contained the study information sheet, informed consent, and a link to indicate their interest in participating. Those who agreed to participate were scheduled into focus groups based on their primary healthcare provider (VA or non-VA). At the start of each focus group session, the research team reviewed the purpose of the research and the informed consent. The research team discussed all elements of the informed consent including the voluntary nature of participation, ability to abstain from answering questions or stop participating at any time, confidentiality, audio recording and transcription, and the collection of demographic information. The focus groups were conducted via the Zoom online meeting platform. An online format was chosen as it allowed for ease of participation for the potential participants, eliminating many participation barriers [27]. Zoom was chosen as it has been well-established as a qualitative tool, and the researchers had access to the subscription [28]. No connectivity issues occurred during data collection. Participants answered questions about their choice of primary healthcare provider, the reasons for said choice, and experiences with their healthcare provider. At the conclusion of each focus group, participants completed a 10-question background survey, and received a USD 25 gift card. Focus group audio recordings were transcribed for qualitative analysis and erased upon transcription completion. Focus groups lasted approximately 45 min. All research procedures were approved by the University of Southern California Human Research Protection Program Institutional Review Board. 

### 2.5. Analysis

To examine the veterans’ healthcare experiences and the factors that influenced their healthcare decision-making, a thematic analysis was conducted. This qualitative research methodology identifies and interprets patterns in the data [29,30] and affords a deeper understanding of veteran healthcare experiences and thought processes by focusing on the data at the experiential level. A systematic approach was employed to ensure the confirmability of the results and interpretations. First, two researchers conducted thematic analysis of the focus groups independently. This analysis began with code identification through a close line-by-line examination of the data. Researchers met periodically throughout the process to compare and discuss their analyses, code identification, and interpretations. Researchers highlighted similarities and differences in identified codes and how they could best be grouped together, developing themes. Agreement was sought following a comparison of individual interpretations, discussing the rationale behind the derivation of the various codes and themes. Agreement on themes was high between the two researchers (97%). Percent agreement was calculated by identifying themes that were found by both researchers. An open discussion reconciled remaining differences for themes not identified by both researchers. This final discussion led to the analysis team identifying and agreeing upon several themes and subthemes for use vs. non-use of VA healthcare. 

## 3. Results

### 3.1. Veterans’ Reasons for VA Utilization

Three themes were identified in association with why veterans chose to receive their healthcare through the VA. These included quality of care, financial benefits, and ease of use. Veterans in this group overwhelmingly described the care they received from the VA as high quality. “The quality of care is top notch”. “You get better healthcare when you go to them [VA]”. This included competent providers who were knowledgeable about their health issues, demonstrated military cultural competency, and cared about veterans’ health. “They know how to hire providers. I’ve had amazing providers throughout an array of different departments”. “I would always choose the VA because of the way you’re treated”.

The most frequent reported reason for VA use by the participants was the financial benefits (i.e., much lower costs). Veterans described limited bills and copays, affordable prescriptions, and the substantially lower costs when compared to outside insurance. In fact, cost was a main driver in most who chose to use the VA for care. “It’s very cost effective going to the VA because we don’t have to pay a copayment”. “Really inexpensive for medication [at the VA] or anything that would’ve cost hundreds if not thousands of dollars in the outside program”. 

The low cost of care included not only free service-connected care but needs outside of those related to the veterans’ time during service. “Even if it had nothing to do with your disability, the ability to pay for it out of pocket or to copay for it was better than what I would’ve paid with private insurance”. “Even if I was seen for an injury or an emergency that was not related to my disability…it was very, very inexpensive”.

For many, care outside of the VA was not even an option due to cost, particularly with their medical issues. “There’s no way I could afford outside care from the VA”. Veterans were often brought to the VA due to significant financial stressors such as the loss of employment or entering higher education. “I got laid off and that’s when I ran out of insurance. I had a medical problem and the VA came in and helped”. “I lost my job. I was unemployed. So cost had a big factor to it”. “I was left without insurance and had a serious injury, hospitalized. My medical bills were over a million dollars and the VA stepped in and took care of everything”. While cost brought many to the VA initially, some described that staying with the VA was also due to the quality and positive experiences.

*“I’m delighted with the care I get at the VA, and it was free, and the idea that I could have competent people who paid attention to me who were concerned, and they ordered tests that they thought needed to be ordered. It was free. I was just delighted to have it and I’d be a fool to do anything else. Before I got involved with the VA, I was self-employed, and I had to buy my own medical insurance for me and my wife. And we felt it, you know, and not have to spend that kind of money. It was good.”*

However, this was not the case for all veterans using the VA. Another group used the VA only due to the cost. “I had to quit my last job due to my injuries. So in terms of cost, I’m kind of forced to go through the VA and utilize them”. “Basically, because it’s free. Honestly, if it wasn’t free. Well, if I had to go outside of the VA, I would probably rack up a million dollars a year based on my health issues”. Others used the VA only in times where non-VA care would be cost prohibitive. “I only use the VA when, basically, it’s absolutely necessary. I’m a hundred percent disabled. When I have to do something like a big procedure, something that would cost a significant amount of money, even with private insurance that I have, then I’ll go through the VA”.

The final theme related to reasons for VA utilization was the ease of use. “I go to the VA and it’s a one stop shop. I get everything I need right there”. Veterans attributed this ease to the ability to transfer medical records, accessible and convenient access to care, the variety of services, and the recent increase in technology resources and tools (electronic records and telehealth). “There are always locations and even clinics closer to my home, other than just the hospital itself”.

*“I guess for myself it was just easier. I’ve had experiences with private insurances and they’re not necessarily any much better in terms of quality. And then once I was service connected with the VA, it just made everything much easier. So, for me, using the VA as my primary insurance is a lot more convenient.”*

### 3.2. Veterans’ Experiences with VA Healthcare

Three themes and three subthemes emerged from the VA users’ experience with their healthcare: overall positive VA experience, challenges to VA care, and gender specific experiences. Overall, veterans who used the VA reported very positive experiences with their healthcare, particularly when referring to the clinical care they received. “I want to emphasize that my [VA] healthcare has been a great experience”. “The VA and the care has been pretty exceptional”. “I love them for everything. I just think they’ve topped any private provider I’ve ever seen”. Many discussed the improvements they had seen in VA care as well. “I think there’s been such great improvement over the past few years that the quality of care has improved dramatically”. “I got out in 2014. I’d say that in the past maybe five years, I’ve noticed a big difference in the quality of the care that I’ve received”.

Despite the reported positive experiences, veterans did describe the challenges they experienced with VA care and how those difficulties often undermined the quality of care. Several subthemes were identified under challenges with care. The first was the lack of continuity of care within VA healthcare. Veterans found that the “constant rotation” of care providers was frustrating and felt that it created difficulty in building relationships with providers. “At the VA, I’ve probably gone through probably 12 [primary care providers] in 2 years”. I’ve gone through three doctors because they keep quitting or going on to better jobs or whatever. And I can appreciate that, I can completely appreciate that, but it’s a little frustrating and disconcerting when my health is at stake”. “I’ve had like five different primary care doctors. And I don’t know why we [the VA] can’t keep some of them because some of them have been really phenomenal”. Besides not having the ability to build rapport with a provider, veterans found it challenging to have to reintroduce their medical history at every appointment. “You’re having to reintroduce yourself every single time, that doesn’t help the situation on either side”. “They see almost an hour later and now you’re having to repeat your whole history for another thirty minutes”. “I would agree, with my primaries, because I’ve had four primaries in the last year and a half, every time I get a new primary, I got to go through all the medical history again”.

Veterans who used the VA attributed the lack of continuity of care to two things. The first was turnover. “I have several doctors tell me personally, I’m leaving next month. Some of them are honest, they tell me like, and they just don’t pay me enough for what I have to do here”. 

“They know how to hire providers. I’ve had amazing providers throughout an array of different departments. And I know that the bureaucracy really pushes them out because these same great providers I’ve had have quit”. “I’d have to agree with respect to the turnover with providers. I’ve been through four primaries in the last year and a half. They keep leaving the VA and going elsewhere”. The second reason for the lack of care continuity described by veterans was the nature of the VA as a teaching hospital. Participants reported the rotation of interns and residents often impacted continuity of care. “Granted this [the VA] is a teaching hospital, which is annoying because you’re constantly having to have a new intern or doctor every two years or a year”. “For me, it’s the residency care that I find it very inconsistent. Every time I’m coming in and there’s a resident there, I have to start from square one. And it can get very frustrating”. In general, the lack of continuity of care was a significant contributor to difficulty with VA care that the veterans described as impacting their time and well-being.

*“There’s a new person every time. And it feels like Groundhog Day, like the movie with Bill Murray. Every time I’m going in there, I’m explaining, I’m like, did you read the chart? Did you read the impressions from the imaging studies? You know, and then it’s like, I’m the one having to instruct them.”*

The next subtheme under care challenges was the bureaucracy around care. “I’d say quality of care [is why I use the VA] but quality of care is often overshadowed by the administrative bureaucracy”. Participants described wait times, lack of communication with the VA within and between departments and specialty care, and general administrative red tape. Frustration with wait times, both for and during appointments, was frequently described by VA users. “Having to wait at the VA, I just take the day and assume it’s going to be hours. I just go with it”. “The only problem is there’s just so many people, getting a follow-up appointment is like months out”. “Just today, I signed up for the women’s therapy group, but the next available appointment was April [over three month wait]”. For some participants, waiting was not always a good option. “I may be seen within the next three to four months, but realistically speaking, I don’t know if I can wait that long”.

*“I have found that access to services like oncology and cancer surgery have been pretty bad. So, I’ve had to use my private insurance and pay more. While I would’ve liked to use the VA and not spend my savings on my private healthcare cost, I did not want to have cancer in my body for an extra four months based on the VA’s wait times.”*

Veterans described the communication barriers within VA care as a source of difficulty. Participants described an environment where “the right hand doesn’t know what the left hand is doing”, most often attributed to situations where veterans needed to seek care across different departments and specialties. “One of the things that I’ve learned through so many years of dealing with the VA is that even the VA doesn’t communicate with themselves”. Lack of internal communication and collaboration often caused delays in care and veterans having to go back and forth between departments. Participants also reported frustrations with the general administrative bureaucracy that hindered care. “It has been a little frustrating sometimes to jump through hoops to get certain things done”. “I get what I want, for the most part but it’s some of the internal bureaucracy that does slow some of that down [needed care] sometimes”. While veterans talked generally of the VA red tape regarding many aspects of care (referrals, medical records, communication, etc.), medical billing was specifically mentioned by many participants as a source of stress. “There were a few times where the VA just kind of threw me a curve ball. And this was mostly because of the medical billing department”. This was described as receiving unexpected charges, not understanding bills, incorrect bills, and challenges in getting those resolved. “Then to [redacted]‘s point with billing and medication, especially with things that aren’t necessarily directed to my service disability, it is correct that the billing is unclear”. Overall, the administrative bureaucracy was described as a significant frustration for veterans and caused barriers to receiving care, often negating the quality of care. “The overall service is great. I’m not doubting that. I was just saying the main barrier to the services is getting in the door and actually getting to it”.

The last subtheme within difficulties with VA care was around advocacy. Veterans reported using a system where great quality of care is provided “but you must work for it”. Participants described having to advocate for themselves to overcome the difficulties described in the challenges to care theme. “It just feels like I have to navigate it sometimes. Like I have to advocate for myself more than the physicians navigate for me”. “Because the VA system is so large, it becomes almost my job to educate. This is how this works. You guys issued this policy and so I’m going to hold you to that”. Participants stressed the importance of being able and willing to engage with the VA to get what they needed. Such engagement took time, effort, and perseverance. “You have to advocate for yourself. If you don’t, nobody else is going to”.

The final theme identified was the women’s experiences within the VA. Veteran women using the VA spoke highly of the expansions and improvements that had been made in women’s care. “Women’s healthcare has expanded and I do appreciate that care actually has been so much better [than] in the past”. Along with the advances in care, women described positive changes in how they were treated and spoken to. “Even how they address us [women] now, every time you come to a clinic, they always say thank you for your service. Before it was like, I was bothersome to them, like I was the problem. But now they acknowledge that I’m a female, I served. I’ve seen the changes”. Women overwhelmingly appreciated the women’s specialty clinics and the positive experiences they have with the care provided at them. “My primary care provider is in the women’s clinic, and I did notice a big difference when I was transferred over that clinic for my primary care”.

*“Once they started building out the women’s clinic, it just made it so much easier. I remember in the beginning, they would put me in a separate room, so I wasn’t in the waiting room with all the men because it was getting awkward. And so in light of that, I personally feel cared for.”*

*“Then the change that happened around 2009 where it’s a woman designated clinic. You don’t have to deal with guys staring at you or asking you what branch your husband was in. That was a big change in 2009 that I think really helped out.”*

Despite the positive experience reported by women, there was some frustration with the time it was taking to improve care for women. “We’ve been here for quite some time. Why are you just making an improvement?” They also described gender specific challenges to receiving care. While veteran women reported positive and competent care within the women’s clinic and/or their primary care provider, historical issues around women’s health in the VA continued to plague outside departments and specialty services. When care was needed outside of primary care or women’s health, women participants continued to experience difficulties such as providers with a deficiency of knowledge around women’s anatomy and healthcare needs, assumptions and disregard around their service, and insufficient equipment. “I get great care for female things at the Women’s Center but once I leave there, things get more disjointed between services”.

“Once I leave the primary care specialty and move over to something like radiology where, you know, we have very specific female anatomy that needs diagnostic tools. It’s super lacking in that area, which I don’t understand”. “They still don’t have certain equipment that females need for testing and treatment”. Finally, women also reported a lack of care and services around fertility and support for single mothers, hoping improvements might be made in both areas.

*“I’ve used the VA for a range of issues for the last 12 years. And some of it was related to having children and fertility. And that’s one area where I felt like the VA did not have access to cutting edge research.”*

*“A lot of us are mothers or single parents or solo parents. I wish they would ask that in our primary because we may need additional assistance that may be hindering our own health based on what our environment or where our situation is living.”*

### 3.3. Veterans’ Reasons for Civilian Healthcare Utilization

Veterans who received healthcare outside of the VA fell into two categories. The first were those who did not have a choice to use VA healthcare due to eligibility issues. The most frequent reason for ineligibility was financial (i.e., veterans made over the income limits to receive care). The second group were ineligible due to not having a service-connected disability. These veterans were either told by the VA that they were not able to receive care, or assumed they could not, due to their lack of rating. About half of the veterans in this group would have chosen to first try the VA if they were eligible. The other half had not been interested in utilizing VA care due to the themes described below. 

The second group of veterans had a choice in where they received their healthcare, and purposefully decided to receive care outside of the VA. Four themes emerged around reasons for the choice, the first, and most predominant, being negative perceptions of the VA. These views of the VA came from personal prior experiences or by word of mouth from veteran friends and family. “I just think the reputation of VA compared to others may not be where it needs to be for me to be comfortable going there”. Several subthemes emerged within negative perceptions including quality of care, VA bureaucracy, and lack of continuity of care. Veterans had concerns about the quality of care the VA could provide. “You have a lot of veterans that feel like they don’t get the care that they deserve”. “I’ve even heard that the care sometimes can be quite poor. I’ve heard horror stories about them just not taking really good care of you”. Veterans also described feeling outside care was superior in quality. “I definitely feel like on the civilian side, I’ve received better healthcare being a veteran from civilian and not federal employees”. “It feels like they care more about us outside of the VA”. 

The second subtheme of negative perceptions of the VA was its administrative bureaucracy. Veterans viewed the VA as filled with red tape that was frustrating and time consuming. “I only went to one [VA appointment] and I was a new patient, just getting there to that point was a lot”. “It’s not a convenient thing [going to the VA]. It’s hard to get an appointment. It takes a long time”. “It’s [the VA] not user friendly, the system is difficult. The waits are long”. “Everybody I’ve talked to says, oh yes, you have to wait forever, the lines are long, and the parking lots are packed. It’s ridiculous”.

*“For me, it was waiting, I think it was three months I waited for my initial appointment after retiring. And then I had so much stuff to talk to the doctor about, that I had to do a follow-up appointment, which took another two months.”*

Veterans did not want to have to navigate the bureaucracy and be forced to advocate for their care. “It’s the time and what they [veterans using the VA] have to jump through hoops to get care. Many years ago, I said, no way. This is going to kill me”. “My few dealings with VA have been frustrating and confusing. So I avoid them”.

The final subtheme within negative perceptions was the lack of continuity of care. Veterans felt the lack of consistent providers with the VA hindered care. “There’s a lack of continuity with doctors. If you have something that is ongoing that needs service over a period of time, you don’t want to have seven different doctors every time you go to see somebody”.

*“It’s [the VA] a teaching hospital. The doctors come and go, you never get the same one. And that’s what I hear from my friends that use the VA. That’s the major concern. They start from step one all the time.”*

The second theme that emerged regarding reasons for non-use of VA was logistical barriers. This was primarily due to distance, with VA hospitals and clinics being too far away. 

“The VA hospital for me is so far away. I have to drive about two hours to get to it. There’s not enough facilities that are close to me to where I can easily access them”. “The VA was too far away from where I’m at”. “Just getting down to [VA city] is a big deal”. When distance was coupled with the administrative challenges, veterans described the VA as just too inaccessible and inconvenient. “It was just a matter of convenience to not utilize the VA”.

The final theme was the lack of dependent care. Veterans wanted to receive care at the same place as their families. “The bigger aspect was my spouse is not military and his medical needs are much greater than mine. I think that it wouldn’t make sense for me to be using the VA when my bigger concern is my dependents”. Having an independent provider felt like an unnecessary burden. “I don’t want to go to the VA and then have to take my children someplace else and have to deal with two different entities. It’s confusing enough trying to deal with one”. 

### 3.4. Veterans’ Experience with Civilian Healthcare

Veterans’ experiences with civilian healthcare were overwhelmingly positive with four themes having emerged during analysis. The first theme, ease of care, related to veterans expressing that their health needs were easily met using their choice of non-VA healthcare providers. “Very smooth, and very awesome. My primary care doctor…He just lines it all up and it happens”. Being able to easily get in touch with providers was a major factor in satisfaction and ease of obtaining needed care. “My healthcare providers are excellent. I can interact with them through a system… they’re very quick to respond. They’re very quick to help”. “They’re easy to get a hold of in contacting the doctor multiple ways. Appointments are quick. They’re effective, they’re efficient”.

The second theme that emerged was high quality of care. Many veterans described the healthcare they received as excellent, and worth it. “I would say you get what you pay for. Even if it [the VA] was cheaper, I think the quality really matters more than anything else”. Some participants highlighted that they felt that their healthcare providers seemed more attentive and caring toward them. “I feel like this sense of warmth and that they actually care about veterans”. “They really took good care of me”. Issues with civilian healthcare were limited, and when veterans did mention them, the difficulties primarily centered around wait times and scheduling. “The only issue I’ve seen throughout the years is that I think maybe they’re enrolling a lot more people. And so appointments used to be a lot easier to get, and now they’re harder to get”.

Freedom of choices was the third theme that emerged. Participants emphasized how significant it was to have flexibility and the option to choose their own healthcare provider. “I’m lucky enough that my employer offers a PPO [preferred provider organization] plan, which gives me a little bit more flexibility to choose the doctors and not have to wait for authorization”. “It comes down to flexibility…if you don’t like a doctor, you can go find a new one. I’m not sure you get that necessarily with the VA”.

The final theme that arose in analysis was cost. Veterans acknowledged that private insurance was more costly than VA care but felt that the quality of care and experience were worth the additional expense. “I would encourage somebody [to use non-VA care] if they can afford it, to do private insurance”. “It’s [civilian care] really good. I’m very thankful, very happy”.

## 4. Discussion

The findings from the current study explored motivations behind VA use and non-use and identified an overarching theme of high satisfaction with the healthcare services received from both veterans who used the VA and veterans who did not. While the study focused on a sample of veterans living in Southern California, the region has the largest population of veterans in the U.S. [31], making it an important veteran population to understand. Furthermore, community-based research on veterans has demonstrated consistency across regions as well as with the national data in identifying challenges experienced by veterans [24,32,33,34,35,36]. It was found that veterans used VA healthcare due to quality of care, financial benefits, and relative ease of use. These positive reports are consistent with the recent literature on the veterans’ healthcare experiences within the VA [21,22]. Although this study was indicative of an overall positive experience using the VA healthcare system, some veterans reported challenges including a lack of continuity of care, difficulty with maneuvering through bureaucratic red tape, wait times, lack of communication between specialties, and limited gender-specific care services. Nearly every veteran who used the VA noted that the costs associated with using the VA was an important reason for doing so. These findings further correspond to the current stated veteran experiences with VA healthcare and reinforce the need to continue improvement in these areas. 

Veterans who did not use the VA healthcare system stated that non-use was either not their choice, as seen through their perceived non-eligibility due to lack of service-connected disability or surpassing the income level limit for fully covered healthcare, or intentionally due to their negative perceptions of the VA, distance from a VA location, or lack of dependent care for scheduled appointments. These findings showed some alignment with previous research that found non-VA users had higher income levels, were younger, and wanted family coverage in their care [37]. Through non-VA providers, veterans experienced positive healthcare service in the form of quality, ease of use, continuity of care, and freedom of choice of practitioner. Reasons people did not use the VA paralleled those aspects of the VA that users found difficult. Findings of this research revealed that many veterans wanted the choice to use care outside of the VA. Furthermore, they revealed high levels of satisfaction with the outside care they received and reported minimal administrative challenges. Expanding options for veterans to receive care outside of the VA should continue to be a priority for U.S. policymakers. 

An important finding for non-VA users was the prominence of veterans who were unable to access the VA due to income limitations, meaning that they had household incomes above the allowable limit for VA healthcare eligibility. This was an unexpected finding and one that warrants further inquiry, particularly as many of these veterans would have liked to use the VA. Income limits appeared to be dependent on where they lived and the number of dependents. In Southern California where these data were collected, income limits for a veteran with two dependents ranged from over USD 99 to 110K total household income. While this income may appear to be high, it is not, especially for a family where both individuals work. While it is important to note that some veterans may be eligible for VA care regardless of income depending on their disability rating, veterans believed that when they entered the service that their healthcare needs would be provided for after they left. That this is not necessarily the case was a surprise to many veterans. Perhaps a model where those in higher income limits pay a fee for services might be more appropriate than denying care outright. While limited research exists on the impacts and outcomes of income limits on VA eligibility, more recent legislation may provide insight into how further expansion might increase access. The recent expansion of the PACT ACT allows for co-pays for veterans who are above the income limit instead of limiting eligibility [38]. 

Despite the advances the VA healthcare system has made since 9/11 and continue to make, there still exists a negative perception of the VA healthcare system among veterans who do not use VA services. While it is historically understood why negative perceptions of the VA might exist, improvements over the last several decades have made the VA a world-class healthcare organization. Research has demonstrated the VA most often performs similarly or better (although not always) than other systems of care when measuring safety and effectiveness [39]. More work is needed to improve perceptions around VA care among veterans and non-veterans alike. The VA might consider a public service campaign to change this negative image among the many veterans who still hold it. As veterans trust each other most when it comes to where they can receive the best services, including veterans in such a campaign would increase the success of such a communication strategy.

Veteran women continue to report VA deficiencies in the ability to meet their primary care needs. Findings are consistent with the previous literature, as veteran women noted experiences of their basic healthcare services as limited or not available [40]. As women comprise over nine percent of the veteran population, with an expectation for that number to double in the next 20 years, continued improvements by the VA for women’s care is needed. The VA must do more now to prepare for the extreme projected demographic shift before services become even more out of reach for women veterans. The expansion of the relatively new women’s clinics throughout the VA is not only necessary for these women, but has greatly improved access, care, and experiences. All VA clinics or hospitals should have, as a minimum, a department or division dedicated to women’s healthcare. More is needed when it comes to integrating gender competent services throughout VA departments. An important finding of this study was that while women felt they received excellent gender specific care within their primary care or women’s clinic, competency around women’s healthcare significantly declined when they required care from outside departments that were not dedicated to women’s health. Women also continue to report a lack of access to and limited appointments for gender specific prevention care. Expansion of women’s services must be at the forefront of the VA healthcare system’s future goals to prepare for the influx of women veterans in the coming years.

The lack of availability of care for family members also kept veterans from using VA services, particularly veteran women. As it is often women who take on the responsibility of healthcare coordination for their family [41], having a separate healthcare system for themselves and for the rest of their family and navigating two healthcare systems felt like an undue burden for many veteran women to handle. These findings are supported by previous work demonstrating that veterans who received care from both VA and non-VA providers experienced significantly more hassles when compared to veterans who received VA care only [42]. This is a reality that has not been fully appreciated by the VA in how healthcare is structured or provided. The VA prides itself on being a “veteran centric” healthcare organization. Perhaps it is time to reimagine the VA as a “veteran family centric” healthcare organization. A place where the veteran and their family can have all their healthcare needs met. This might involve expanding the Mission Act or allowing veteran family members to receive healthcare at VA facilities. Future research might assess the need for dependent care and associated healthcare service utilization and provide the necessary evidence for the VA to pursue an expansion of family benefits.

Previous attempts to address veteran reported feedback or noted areas of potential improvements within the VA healthcare system have often been met with resistance from the VA. Although necessary for service improvement, such feedback is often dismissed by some within the VA as either being framed as complaints from a few disgruntled veterans, with an overarching theme of the impossibility to appease every single service member, or the assertation that the veteran just does not know where to go to obtain the needed support. However, none of the VA findings reported here about the challenges veterans encounter are new. Such feedback has been heard for as long as the VA has existed, going back over half a century. Similar administrative burdens in Medicaid and Medicare have also been found to reduce access to care [43,44]. Indeed, two phrases are often spoken by senior leaders within the VA. The first being “This isn’t your grandfathers’ VA”. The meaning here being that the VA has changed and is changing. This is certainly true, yet findings here indicate change is happening slowly. The other phrase often heard is “If you have been to one VA, you have been to one VA”. This saying denotes that every VA is different with different priorities, different services, and with different approaches to healthcare. These phrases, which we believe broadly represents the VA culture, means that change will be slow and uneven across the nation. Continuing to examine how this impacts veterans’ health, how improvements can be made more quickly, and providing stopgaps for improvements that will be more long-term will be essential to caring for our nation’s veterans. 

As with all research, several limitations must be noted. As the study was qualitative, the results represent the experiences of those veterans who participated in the study and are not intended to be generalizable. Additionally, this research examined non-VA users as one group. While this was expected, as the purpose of the study was to explore how veterans chose VA or non-VA care, it does limit any opportunity to understand nuances between different types of non-VA care. While researchers were able to explore gender differences in care, the group size did not allow for examining other demographic differences that may impact choices in care. Despite these limitations, the findings provide insightful data into what drives healthcare decision-making in veterans. 

Future research may build upon these findings while also addressing some of the noted limitations. Further exploration of gender differences in healthcare decision-making and the experiences of veteran women will be important to address the needs of this growing population. While much progress has been made within the VA in regard to women’s healthcare, continued advocacy supported by data will be required to achieve further improvements. Research might also consider looking into any demographic differences in use, choices, and experiences in veterans’ healthcare. Survey data might also provide additional insights into variables that are unable to be explored through qualitative analysis, particularly demographic, background, and societal factors that may impact decision-making. Longitudinal data could also provide information on shifts in healthcare decision-making. As healthcare options outside of VA care continue to be important for the veteran community, further exploration into the use of, experiences with, and innovations within civilian care providers will be essential. 

## 5. Conclusions

The findings of this research provide new insights into an important gap within the veteran healthcare literature, exploring the drivers of healthcare decisions and current experiences within both VA and civilian healthcare providers. The qualitative approach provides a novel in-depth look at veterans’ decisions and experiences with healthcare. Overwhelmingly, veterans were happy with the care they received regardless of whether it was provided by the VA or civilian providers. Veterans who used the VA made that choice most often due to the quality of care, low cost, and ease of use. However, veterans continue to acknowledge the challenges associated with VA care including the lack of continuity of care, administrative bureaucracy, having to advocate for care, and limitations in gender specific care. Veterans who chose to receive care from a civilian provider most often did so due to negative perceptions or experiences with the VA and not wanting to have to deal with the administrative challenges associated with VA use. Continuing to understand veterans’ choices and experiences with healthcare is essential to meeting the care needs of our nation’s veterans.

## Figures and Tables

**Table 1:** Focus group interview protocol.

Questions Primary Care VA ^1^
What are the factors that led to your decision to choose the VA as primary source of healthcare? What keeps you using the VA?
What have your experiences been like using the VA?
Do you feel your healthcare providers are sensitive to your needs and experience as a veteran? For the women in the group, do you feel your healthcare providers are sensitive to your needs and experience as a woman? What do you think the VA does well? What do you think the VA needs to improve on? What are the biggest challenges to accessing care? One of the biggest barriers to using the VA in the original study was the perception that care is not as good at the VA. Have you heard this? Where do you think that stigma comes from? What if anything might make you switch to non-VHA care? Would you encourage other veterans to use the VA?
**Questions Primary Care Civilian Provider** ^1^
Can we start with everyone who is comfortable indicating where they get their primary care from? How many from employer insurance? From the choice program? A program such as Medicare, Medicaid or Covered California? Somewhere else? What are the factors that led to your decision to choose your primary source of healthcare? What have your experiences been like using your primary source of healthcare? Do you feel your healthcare providers are sensitive to your needs and experience as a veteran? For the women in the group, do you feel your healthcare providers are sensitive to your needs and experience as a woman? What do you think your primary care provider does well? What do you think they need to improve on? What are the biggest challenges to accessing care? Is there anything specific about the VA that made you choose not to use VA care as your primary provider? One of the biggest barriers to using the VA in the original study was the perception that care is not as good at the VA. Have you heard this? Where do you think that stigma comes from? What if anything might make you switch to using VA care? Would you encourage other veterans to use your source of healthcare?

^1^ All questions were asked of each focus group.

**Table 2:** Focus groups.

Group	Number of Participants (*n* = 59)
VA Users Group 1	10
VA Users Group 2	10
VA Users Group 3	8
VA Users Group 4	5
Non-VA Users Group 1	8
Non-VA Users Group 2	4
Non-VA Users Group 3	4
Non-VA Users Group 4	10

**Table 3:** Demographic characteristics of sample.

	Percent
Age	
18–29	1.7
30–39	22.4
40–49	19.0
50–59	20.7
60–69	22.4
70 and older	13.8
Race	
American Indian or Alaska Native	---
Asian	6.9
Black or African American	15.5
Native Hawaiian or Other Pacific Islander	---
White	72.4
Other	10.3
Hispanic, Latino or Spanish Origin	
Yes	25.9
No	74.1
Gender	
Man	56.9
Woman	38.0
Non-Binary or Third Gender	1.7
Prefer not to say	1.7
Prefer to self-describe	1.7
Highest Education Level	
High School Diploma	1.7
Some College	15.5
Associate’s Degree	8.6
Bachelor’s Degree	25.9
Master’s Degree	36.2
Doctorate Degree	6.9
Other	3.4
Missing	1.7
Marital Status	
Single	19.0
Married	56.9
Divorced	20.7
Widowed	3.4
Branch of Service	
Air Force	17.2
Army	27.6
Marine Corps	25.9
Navy	29.3

## Data Availability

The data that support the findings of this study are available on request from the corresponding author. The data are not publicly available due to privacy or ethical restrictions.
